# Local hyperthermia decreases the expression of CCL-20 in condyloma acuminatum

**DOI:** 10.1186/1743-422X-7-301

**Published:** 2010-11-04

**Authors:** Xiaoqin Wang, Xing-Hua Gao, Yuxiao Hong, Xiaodong Li, Hong-Duo Chen

**Affiliations:** 1 Department of Dermatology, No.1 Hospital of China Medical University, Shenyang 110001, PR China; 2 Department of Dermatology, Shengjing Hospital of China Medical University, Shenyang 110004, PR China

## Abstract

**Background:**

Local hyperthermia has been successfully used in the treatment of viral warts. However, the mechanism of action has largely remained unclear. CCL-20 (also known as MIP-3α) is the most potent chemokine for recruitment of Langerhans cell (LC) precursors into the skin. CCL-20 expression can be increased by TNF-α and IL-1α. The effects of local hyperthermia on the mRNA expressions of CCL-20, TNF-α, IL-1α have been investigated in both condyloma acuminata (CA) and normal skin. Under an organotypic culture condition, fresh CA and normal skin were subjected to surface heating at 37°C, 42°C and 45°C for 30 mins, respectively.

**Results:**

The mRNA expressions of CCL-20 and IL-1α in CA specimen were significantly higher than those in normal skin. Local hyperthermia at 42°C and 45°C significantly decreased the mRNA levels of CCL-20 and IL-1α, as compared with the control groups (p < 0.01). The decrease of CCL-20 was well correlated with that of IL-1α. The expression of TNF-α in CA remained unchanged in spite of the temperature variation. Local hyperthermia at 45°C concomitantly increased the mRNA expression of CCL-20 and IL-1α in normal skin.

**Conclusions:**

Our study suggests that hyperthermia decreases the expression of CCL-20 with concomitant decrease in IL-1α, and reduce the number of Langerhans cells in HPV infected skin.

## Background

Human papillomavirus (HPV) is a group of circular-DNA viruses that could induce proliferation and, under certain circumstance, the malignant transformation of epithelial cells in humans [1]. HPV infection of human skin or mucosa results in a variety of clinical entities, *e.g. *common warts, plantar warts, condyloma acuminata (CA), etc, primarily based on the anatomical sites and the invading types of HPV. Most of HPV infected lesions resolve spontaneously within years, while some are recalcitrant.

Hyperthermia, either within or above fever range, has been reported to treat a number of cancers with various efficacies [2]. Local hyperthermia has been successfully implemented in the treatment of HPV- infected skin lesions [3-5], though the mechanisms of action remain largely unknown.

Langerhans cells (LCs) are dendritic cells that reside in the epidermis, and are believed to be professional antigen presenting cells. CD1a is a relative specific marker of epidermal LCs. We previously demonstrated that local hyperthermia decreased the number of CD1a^+ ^LCs in epidermal compartment of normal skin and CA. The decrease in LCs was temperature dependant and was more pronounced in CA than in normal skin. The decrease in number of epidermal LCs was mostly attributed to their migration to regional lymph nodes, where LCs encountered, possibly prime the T cells. In addition, hyperthermia was found to down-regulate the expression of CCR6 and up-regulate the expression of CCR7 in emigrated CD1a^+ ^cells, a pre-requisite for the immigration of LCs away from the epidermis [6]. Homeostasis of epidermal LC could be readily reached after the stimuli, as signals are generated to recruit LC precursors into the skin, maintaining the epidermal LC population [7]. Among an array of chemokines, CCL-20 (also known as MIP-3α) is the most potent chemokine for recruitment of LC precursors into the skin [8,9]. CCL-20 expression was reported to be increased by TNF-α and IL-1α in oral squamous cell carcinomas, intestinal epithelial cells, and keratinocytes [8,10-12].

Using a modified skin organ culture protocol, we investigated the effects of local hyperthermia on mRNA expression of CCL-20, TNF-α and IL-1α in both normal skin and CA, and further explored the possible mechanism of local hyperthermia on immigration of epidermal LCs.

## Methods

### Patients and specimens

Twelve patients (4 male and 8 female) with clinically diagnosed CA were enrolled into the study. Enrolled patients had not undergone any prior anti-wart treatments. Patients with documented systemic disease(s) were excluded from the study. Biopsies were obtained from pudendum. Four skin specimens from patients undergoing genital plastic surgery were obtained as the normal controls. Informed consent was obtained from the patients and controls. This study was approved by the Ethic Committee of China Medical University.

### Hyperthermia device and local hyperthermia protocol

As previously described [13], a patented (patent name: a hyperthermia device; patent No.: ZL 2007 2 0185403.3; patent holder: China Medical University) local hyperthermia device was used in the study. The heat generated by the device acted on skin surface without direct contact and the surface temperature was readily adjusted to the point as desired. A skin organ was cultured as previously reported [14]. Briefly, approximately 1 × 1 cm^2 ^of fresh CA (n = 12) and normal skin specimens (n = 4) were cut into 4 equal portions. One of them was frozen and stored at -70°C. Three pieces were separately placed in culture dishes, with dermal side down in the media (RPMI with 10% FBS, 2 mM glutamine, 100 U/ml penicillin, 100 μg/ml streptomycin, 55 mm β-mercaptoethanol), surface exposed to the air. The exposed surface was subjected to local heating at surface temperatures of 37°C, 42°C and 45°C, respectively, for 30 mins. Then they were fully submerged in culture media and incubated at 37°C for 12 hr with 5% CO_2_. At the end of experiment, the specimens were embedded in Tissue-Tek(r) (OCT compound, Sakura, USA), frozen in liquid nitrogen and stored at -70°C for further analyses.

### Fluorescent Real-time (Quantitative) PCR

The Fluorescent Real-time (Quantitative) PCR was performed as described [13,15]. Briefly, total RNA was extracted from specimens using Trizol reagent (Gibco) and reverse transcribed with First Strand cDNA Synthesis Kit (Tiangen). Real-time PCR was conducted on a Roter-Gene 3000 Detector System (Corbett Research, Australia) with the QuantiTect SYBR Green PCR RealMasterMix Kit (Tiangen, FP202). Relative quantitative analysis was performed using the rotor-gene software (Version 5.03). The PCR conditions were as follows: Initial denaturation at 95°C for 2 min, followed by 40 cycles of denaturation at 95°C for 15 s, annealing at 57°C for 30 s, extension at 68°C for 45 s. All assays were performed in triplicates. The primers used in this study were designed according to the Genbank sequences: GAPDH (forward, 5'-GAA GGT CGG AGT CAA CGG AT -3'; reverse, 5'-CCT GGA AGA TGG TGA TGG G-3'); CCL-20 (forward, 5'-CTG CGG CGA ATC AGA AGC-3'; reverse, 5'-GCA AGT GAA ACC TCC AAC CC-3'); TNF-α (forward, 5'-ACA CCA TCA GCC GCA TCG-3'; reverse, 5'-GCG TTT GGG AAG GTT GGA T-3'); IL1-α (forward, 5'-TTG TAT GTG ACT GCC CAA GAT G-3'; reverse, 5'-AGA CCT ACG CCT GGT TTT CC-3').

### Statistical analysis

Data were analyzed with SPSS 13.0 software. The differences in the expression of CCL-20, TNF-α, IL-1α mRNA were analyzed by ANOVA and independent-sample t test. The correlation between the expressions of CCL-20, TNF-α, IL-1α mRNA was analyzed by two variable correlation analyses. A *p *value of <0.05 was considered to be statistically significant.

## Results

**Figure 1:**
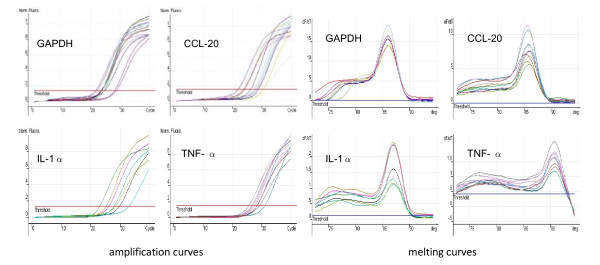
**Real-time PCR was conducted on a Roter-Gene 3000 Detector System**. These were the amplification and melting curves of GAPDH, CCL-20, TNF-α and IL-1α in CA and normal skin at different temperatures.

The RT-PCR amplification and melting curves were shown in Figure 1.

### The mRNA expression of CCL-20, TNF-α, IL-1α in fresh CA and normal skin

As shown in Table 1, in fresh untreated normal skin, the mRNA expression of CCL-20, TNF-α, IL-1α was very low. The mRNA expression of CCL-20 and IL-1α in CA was significantly higher than those of normal skin (*p *< 0.01). The mRNA expression of TNF-α was also high in CA, as compared with fresh normal skin (*p *< 0.05).

**Table 1:** The mRNA expressions of CCL-20, TNF-α, IL-1α in CA and normal skin subjected to different hyperthermia temperature.

	fresh		37°C		42°C		45°C	
	
	CA	Normal skin	CA	Normal skin	CA	Normal skin	CA	Normal skin
CCL-20	1037.8 ± 139.8^##^	0.96 ± 0.11	899.3 ± 198.9^##^	0.86 ± 0.23	128.1 ± 45.4**^##^	1.06 ± 0.38	140.2 ± 36.3**^##^	2.41 ± 0.7*
TNF-α	2.43 ± 0.54^#^	1.05 ± 0.19	1.47 ± 0.41	1.09 ± 0.08	1.73 ± 0.32	0.68 ± 0.17	1.24 ± 0.57	1.16 ± 0.24
IL-1α	122.48 ± 51.6^##^	1.09 ± 0.17	78.8 ± 14.46^##^	0.95 ± 0.15	23.5 ± 6.39*^##^	0.74 ± 0.13	7.94 ± 1.44*	5.08 ± 2.63*

Compared with fresh and 37°C group, the expressions of CCL-20, IL-1α mRNA were significantly decreased after local hyperthermia at 42°C and 45°C in CA; In normal skin, CCL-20, IL-1α mRNA expressions increased significantly after hyperthermia at 45°C compared with those in fresh and 37°C group; The expressions of TNF-α mRNA remained unchanged in spite of the temperature variation in normal skin and CA. (*p < 0.05; ** p < 0.01 versus untreated and 37°C group); The expressions of CCL-20 mRNA in CA were all significantly higher than those in normal skin at different temperatures. The expressions of TNF-α mRNA in CA were higher than that in normal skin only in untreated group. The expressions of IL-1α mRNA in CA were significantly higher than those in normal skin except for 45°C group (^#^p < 0.05; ^## ^p < 0.01 versus normal skin);

### Effect of local hyperthermia on mRNA expression of CCL-20, TNF-α, IL-1α in CA

As shown in Table 1, the mRNA expression of CCL-20, IL-1α was slightly decreased in specimens treated at 37°C than those of untreated specimens, while no statistical difference was detected (p > 0.05). Compared with untreated fresh specimens and those treated at 37°C, mRNA expression of CCL-20 and IL-1α was significantly decreased after local hyperthermia at 42°C and 45°C (all p < 0.01). The mRNA expression level of IL-1α was positively correlated with that of CCL-20 mRNA in CA (r = 0.57, *p *= 0.034). TNF-α mRNA remained unchanged despite the different local temperatures applied (p > 0.05).

### Effect of local hyperthermia on mRNA expression of CCL-20, TNF-α and IL-1α in normal skin

As shown in Table 1, the mRNA expression of CCL-20, TNF-α, IL-1α was in low levels in untreated normal skin. The low expression levels were not apparently affected by treatment with local hyperthermia of 37°C and 42°C (p > 0.05). However, after hyperthermia treatment at 45°C, mRNA expression of CCL-20 and IL-1α was increased significantly, as compared with those of untreated and treated at 37°C (p < 0.05, respectively). The mRNA expression of TNF-α in normal skin remained unchanged after hyperthermia treatment at 45°C.

## Discussion

Tissue injury, microbial infection, contact allergens, etc. provide danger signals, leading to a local production of proinflammatory cytokines that induce LC mobilization to the lymphoid tissue. At the same time, signals are generated to recruit LC precursors into the skin and maintain the epidermal LC population [7]. It has been documented that the number of LCs in epidermis decreased in HPV infected skin [16]. However, the significance of LCs populated in the skin under different conditions has been hotly debated. Fushimi T et al showed that CCL-20/MIP-3α transgene attracted dendritic cells to established murine tumors and suppressed tumor growth [17]. Bonnotte et al [18] indicated that the high density of DCs in the tumor site was not a sufficient condition to induce an immune response. Furthermore, this attraction of immature DCs may always have an adverse effect by inducing a tolerance to the tumor cells. We have recently demonstrated that local hyperthermia decreased the number of epidermal LCs in a temperature dependant manner, in both normal and HPV infected skin. It was also noted that expression of CCR-7 increased and CCR6, decreased on emigrated CD1a+ cells in temperature dependant manner [6]. Hyperthermia induced immigration of LCs away from the epidermis were recovered in about one to two weeks [19]. CCL-20 is a CC chemokine constitutively expressed in tissues such as liver, lung, follicle-associated epithelial cells. In skin, CCL-20 is mainly expressed in keratinocytes, skin fibroblast, microvascular endothelial cells and dendritic cells [20,21]. The ligand-receptor pair CCL20-CCR6 is responsible for the chemoattraction of immature dendritic cells, effector/memory T-cells and B-cells [22]. CCL-20 was weakly expressed in normal human skin but was strongly augmented in some inflamed diseases, such as atopic dermatitis and psoriasis [10,11]. In vitro studies showed that following proinflammatory stimuli, both high- and low-risk HPV E6 and E7 inhibited CCL-20 transcription, resulting in suppression of the migration of immature Langerhans precursor- like cells [23,24]. The expression status of CCL20 in clinical specimen has not been documented. In the present study, we detected high expression of CCL20 mRNA in CA, as compared with normal skin, a result contradictory to the *in vitro *observation. It was speculated that: (1) HPV E6 and E7 genes were not active in highly differentiated layers of epidermal keratinocytes; (2) uninfected keratinocytes or infected keratinocytes without HPV E6 and E7 expression produced CCL20 locally; (3) other cellular types, such as Langerhans cell, fibroblast, endothelial cells or infiltrating lymphocytes produced CCL20 locally. Local hyperthermia at 42°C and 45°C for 30 min reduced the mRNA expression of CCL20 to about 6- and 7- folds, respectively. We previous showed that local hyperthermia under certain condition was able to reduce the mRNA expression of CCR6 on immigrated CD1a+ Langerhans cells, and there was a concomitant decrease of epidermal LCs [6]. As the ligand-receptor pair CCL20-CCR6 is responsible for the chemoattraction of immature dendritic cells to the epidermis, we conclude that instantaneous local hyperthermia is an unfavourable condition to attract LCs into epidermis, instead it provides a favourable milieu to drive LCs away from the epidermis. Paradoxically, local hyperthermia at 45°C increased CCL-20 mRNA to about 2.5 fold in normal skin, as compared with untreated specimens. The increase in expression was marginal and its significance needs further study.

It has been shown that upon stimulation with IL-1α and TNF-α, CCL-20 was produced by keratinocytes at high level [10,11]. In the present study, we observed concomitant decrease of IL-1α and CCL-20 in CA with concomitant increase of IL-1α and CCL-20 in normal skin. In addition, CCL-20 expression appeared to be positively correlated with IL-1α under hyperthermia conditions. Either in CA or in normal skin, TNF-α expression remained unchanged in spite of the hyperthermia conditions. The result was partly consistent with the finding by others [25] that heating had no effect on the expression of TNF-α. Local hyperthermia affects several other cytokines. Zhu et al [26] showed that hyperthermia at 42°C and 45°C was able to induce a significant increase in the transcriptional expression of IFN-α, IFN-β and IFN-γ, in a temperature-dependent manner in CA, but not in normal skin. The mechanisms of local hyperthermia were complicated. Different studies indicated that the combination of different pathway is probably contributory to eliminating the warts.

## Conclusions

Our study suggests that hyperthermia decreases the expression of CCL-20 with concomitant decrease in IL-1α, and reduce the number of Langerhans cells in HPV infected skin.

## List of abbreviations used

CA: Condyloma acuminata; CCL: CC chemokine ligand; CCR: CC chemokine receptor; HPV: Human papillomavirus; IL: Interleukin; LC:Langerhans cell; MIP: Macrophage inflammatory protein; TNF: Tumor necrosis factor.

## Competing interests

The authors declare that they have no competing interests.

## Authors' contributions

XW carried out the studies and drafted the manuscript. XHG conceived of the study, and participated in its design and coordination and helped to draft the manuscript. YH participated in the statistical analysis. XL participated in the collection of specimens. HDC participated in the revision of the manuscript. All authors read and approved the final manuscript.
